# A Treatise on Dislocations and Fractures of the Joints

**Published:** 1842-07

**Authors:** 


### Art. II.

*A Treatise on Dislocations and Fractures of the Joints.* By Sir Astley Cooper, Bart. f.r.s., Serjeant-surgeon to the King, &c. Edited by Bransby B. Cooper, f.r.s., Surgeon to Guy's Hospital. —*London*, 1842. 8vo, pp. 576.

The announcement of a new edition of one of Sir A. Cooper's greatest works, edited by his nephew Mr. B. Cooper, is calculated to excite high expectations on the part of the profession. The original work is unquestionably the most valuable contribution that has ever been made to the department of surgery of which it treats, and though not all perfect, (what work ever was, or will be so?) it must yet long remain the classical authority respecting these injuries. It would, however, be absurd to deny that many valuable additions have been made to this branch of knowledge since the first publication of this treatise; and indeed it would be a very questionable compliment to the illustrious author to maintain such a proposition. The happiest result of a real addition to the existing stock of knowledge on any subject is, that it almost certainly leads to further improvements in the same direction; an impulse is given to enquiry, and the newly-opened paths of investigation are explored, with the almost certain consequence of placing on record much valuable matter, which would previously have escaped observation, or been neglected as unimportant. We accordingly know that since the publication of Sir A. Cooper's work on Dislocations, surgeons throughout the world have constantly compared the results of their practice with the doctrines laid down in that incomparable book, and the result has been that additional information has been obtained respecting many points of considerable importance. A new edition of this great work was, we therefore think, called for. Such an edition as we would desire to see could not be expected from the hands of the author. He was indeed, *rude donatus,* and might well depute to another the task of producing an annotated edition presenting the comparatively slender yet absolutely important corrections and additions rendered requisite by the multiplied experience of numerous observers.

If the present volume was a mere reprint we should not consider it necessary or right to notice it in this review, but we are told (Preface, pp. ix, x,) that much new matter, derived from Sir A. Cooper himself, selected from the editor's own practice, and contributed from various sources, has been added to this edition. But above all we find that this is an annotated edition, being accompanied with notes, some intended to illustrate, others to correct the doctrines contained in the text; and we consequently infer that the editor designs the present volume not as a mere exposition of Sir A. Cooper's experience and opinions, but professes also to exhibit the existing state of knowledge and opinions respecting the various subjects of which it treats. We therefore feel ourselves called on to examine how far the promises thus implied have been fulfilled, and shall at the same time lay before our readers the marrow of the more important new matter that is incorporated in the present edition. We must not be understood as undertaking to notice the entire work in detail; it will be only necessary to advert to a few of the more prominent subjects, from which as a specimen, an opinion may be formed of the manner in which the whole is executed.

We must first, however, briefly advert to one or two points, which we think require at least a passing remark. We are told (Preface, p. xi.) that Mr. B. Cooper, in consequence of having been much occupied in writing the life of Sir A. Cooper, and also in attending to his professional avocations, has deputed to Mr. Druitt the task of "arranging the new matter, and generally preparing this edition for the press." Had Mr. Druitt's co-operation been obtained merely with the view of rendering the editorial comments more copious and perfect, we should be far indeed from finding fault, as we are well aware how valuable would have been that gentleman's assistance in such a task; but we do find fault with the reason assigned by Mr. Cooper for his having thus sought assistance. Sir A. Cooper contrived, amidst professional occupations of perhaps unexampled extent, to produce the work which Mr. B. Cooper cannot find leisure to edit. We trust that in any similar task which he may undertake with respect to any other of the works of his illustrious uncle, he will not allow his time to be occupied, or his attention distracted by any concurrent literary undertaking. We say this the rather because the present volume betrays signs of carelessness, which, though not very important, nor really detracting from the value of the work, are yet blemishes which would have been better avoided, and which a very moderate amount of editorial attention would have sufficed to obviate.

We have also to regret that the editor has not presented us with a strictly accurate transcript of the original text. We are told (Preface, p. x.) that "it has been thought necessary, in some few instances, to condense the original," and this for the sake of admitting the new matter, without swelling the size of the volume beyond a certain limit. We are happy to be enabled to assure our readers that the "condensations" to which we object are on the whole few and unimportant, and can hardly be considered, as impairing the authority of the text. But we must add that they would have been much better altogether avoided. In some instances passages eminently characteristic of Sir A. Cooper's style and manner have been omitted, as an example of which we need only cite the suppression of the most graphic "instance of mistake" related at p. 2 of the 4to edition. We shall probably, however, have occasion hereafter incidentally to advert to this subject, and need not dwell further on it at present.

*Dislocations in general.* Important as is the subject of this chapter, it does not on the present occasion call for any very extended remarks; the editorial notes are more numerous on this than on any other chapter in the work, and we think we may fitly refer to some of them as bearing out the observations we have above made, and showing that the occupation of the editor's time by other avocations has led to some inaccuracies and oversights, which would have been avoided had he devoted his undivided and serious attention to his undertaking.

The very first editorial remark in the present volume (note, p. 3) evinces, we think, the haste or carelessness of which we complain. Sir A. Cooper in the text makes the important practical remark, that, in the first moments after dislocation, considerable motion often remains, and the position of the limb is not so determinately fixed as it afterwards becomes: in evidence of this position a clinical fact is as usual immediately adduced, and the author says,

"I have seen a man brought into Guy's Hospital who, but a few minutes before, had the thigh bone dislocated into the foramen ovale, and I was surprised to find, in a case otherwise so well marked, that a great mobility of the bone still existed at the dislocated part; but in less than three hours it became firmly fixed in its new situation by a permanent, or, as it is called, *tonic* contraction of the muscles."

The following is the editor's note on this passage:

"This extent of mobility would not have been present in any other dislocation of the hip-joint, but into the foramen ovale; so that the comparative greater mobility in this luxation forms one of its diagnostic marks."

We must say, that this comment combines the twofold demerit of being calculated to weaken the force of a most important remark contained in the text, and of inculcating a false and most unpractical doctrine respecting dislocation of the femur into the foramen ovale. The truth is, and we are sure that no one is better aware of the fact than Mr. Bransby Cooper, that a very considerable extent of mobility may remain for some time after perhaps any dislocation of an orbicular joint. We have ourselves seen two cases of dislocation of the femur on the dorsum of the ilium in which this phenomenon was remarkably obvious, and in one of these cases the surgeon who first saw the patient entertained doubts as to the real nature of the accident, because of the great extent of motion which the limb admitted of, though the symptoms were otherwise most strikingly marked. But we need not insist further on this point: we should not indeed have adverted to it, were not the comment on the text calculated to weaken, indeed to do away with the force of a precept of great importance, as regards both the diagnosis and treatment of certain dislocations when seen shortly after the receipt of the accident. The second error contained in this annotation is perhaps of less importance, but yet requires correction. A "comparative greater mobility," to borrow the editor's phraseology, does *not* form one of the diagnostic marks of dislocation into the foramen ovale; and had but a moderate degree of attention been paid to the text whereon the editor commented, this mistake, the result obviously of sheer haste, could not have been committed; for we are told that the limb, at first unusually moveable, "in less than three hours *became firmly fixed in its new situation*." No doubt, in dislocation into the foramen ovale, the limb admits of abduction and flexion to a considerable extent; but the editor cannot mean to imply that Sir A. Cooper was ignorant of this fact; the text of course conveys that the motions of extension and abduction, the motions *usually limited*, in this dislocation were extraordinarily free in the first instance.

At pp. 12-13 is recorded a case in which a dislocation of the femur into the ischiatic notch was reduced with unusual facility, an event at the moment attributed to the relaxation caused by the presence of nausea. The patient died, however, the following day, when the jejunum was found to be ruptured. To this case the editor appends the following note:

"This case exemplifies the necessity of carefully investigating every circumstance connected with every injury before pronouncing our prognosis or proceeding to treatment. It is evident that the facility with which this dislocation was reduced depended on the mortal injury of the intestines."

Surely the editor did not imagine that this note was his own. If we turn to p. 22 we shall find Sir A. Cooper himself saying,

"That the muscles are the chief opponents to reduction is strongly evinced by those cases in which the dislocation is accompanied by injury to any vital organ....... *Thus, in the case already mentioned, of the man who had an injury to his jejunum and a dislocation of his hip, the bone was reduced with little difficulty.*"

And, at p. 33, Sir A. Cooper impresses on us that it now and then happens that the surgeon's attention is so abstractedly directed to the obvious accident, that he overlooks some important concomitant injury. "I need not remark, then, on the necessity of such examinations being invariably made in every case of severe local injury."

In p. 13 we find a "condensation" which leads to a misrepresentation of Sir A. Cooper's opinion respecting the possibility of the occurrence of dislocation of the vertebræ without concomitant fracture. In the 4to edition, p. 14, it is stated that the accidents which have been called dislocations of the spine "are not true dislocations of the spine, *excepting those of the upper cervical vertebræ*." In the present edition the words printed in italics are omitted; why, we cannot pretend to say. It is remarkable, however, that, coupled with this omission, we have an editorial note, informing us that dislocation of the vertebræ may occur. The fact is, that Sir A. Cooper never denied the possibility of luxation of the cervical vertebræ, as may be further seen by referring to p. 500 of the 4to edition, or p. 526 of the present edition. He merely says that he has never witnessed such an accident. The note in question is, however, a very proper addition to the text; and we have only to remark, that it would have been still more so had it supplied the deficiency of observed cases in the original by a fuller reference to the experience of other surgeons. Thus, we are told that "Boyer has related cases in which dislocation of the atlas from the vertebra dentata has occurred, and death was the immediate result." No doubt he has; but the experience of J. L. Petit and other eminent surgeons who have observed the same accident should have been alluded to, and the history of other luxations of the cervical vertebræ should, we submit, have been given in a more extended and satisfactory form. We were particularly surprised to find that no allusion is made in this note to the remarkable case of dislocation of the second cervical vertebra—the most remarkable case on record of this class of injuries, detailed in the Gaz. Méd. de Paris, 1840, No. xlii. p. 657, et seq., and which was reduced after the lapse of seven months by M. Jules Guerin. It is true that the nature of the accident may be questioned, as there was *no dissection;* but as MM. Marjolin, Bouvier, Sanson, and Guerin all agreed that the case was one of dislocation of the second cervical vertebra on the third, it was surely worthy of being mentioned in a note which professes to supply a lacuna in the MAGNUM OPUS on this class of injuries.

We have hitherto noticed editorial comments which we conceive to be uncalled for or incomplete; and we have now to advert to the absence of comment where we conceive it might have been useful. At p. 17 Sir A. Cooper says, " I have read of dislocations of the hip in children, but their history is that of diseases of the hip-joint, in which the dislocation has arisen from ulceration;" but yet at p. 37 is recorded the case of a child aged seven, admitted to Guy's Hospital labouring under traumatic dislocation of the femur on the dorsum of the ilium; and here, by the way, we must again complain of a suppression which injures the authority of the text; this case is we believe unique, at least we are not aware of any example being recorded of a similar accident having occurred in a child of such tender age; a reference to the present edition would lead to the supposition that this case had occurred in Sir A. Cooper's own practice, and was verified by his own personal observation; and yet we find, on reference to the 4to edition, p. 50, that Sir A. Cooper *did not see the case*, that it was communicated to him by " Mr. Daniel, one of Mr. Lucas's dressers," who " relieved the limb in the presence of many of the students." We do not for a moment mean to insinuate any suspicion as to the authenticity of the case, but we do maintain that this is not the way in which the materials for the history of surgery should be written. We have no note from the editor on this important matter. The few analogous cases of dislocation of the femur in early life should, we take it, have been appended in a note; at least the recent case recorded by Mr. Norris, of the Pennsylvania Hospital, of dislocation of the femur on the dorsum of the ilium, in a boy aged eleven, might, we presume, without any very extended research, have fallen under the notice of the editor.

Previously to the publication of Sir A. Cooper's Treatise on Dislocations, in the event of a bone being both dislocated and fractured, the rule of practice was, in accordance with the recommendation of J. L. Petit, to defer any attempt at reducing the dislocation till the fracture was consolidated; reduction consequently was very rarely accomplished in these cases. Sir A. Cooper, on the contrary, inculcated the propriety of endeavouring to reduce the dislocation without delay, provided the fracture occurred at a sufficient distance from the displaced extremity of the bone. This is perhaps the only practical rule in Sir A. Cooper's entire work which is not based on and illustrated by clinical observation; it is indeed not a little remarkable that not a single case is recorded by Sir A. Cooper of dislocation and fracture of the same bone in which the practice he recommends was adopted; but two cases of fracture with dislocation of the femur are mentioned. In one (p. 49) the fracture was allowed to unite, and in five weeks Mr. Badley reduced the dislocation. In the second case (p. 50) the dislocation remained unreduced. But one case of fracture and dislocation of the humerus is mentioned (p. 382), the fracture having united, a fruitless attempt at reduction was made six weeks after the accident. The value of the rule of practice inculcated by Sir A. Cooper has been nevertheless amply established, and we think the editor should, when sending an annotated edition into the world, have cited some of the cases which exemplify the sagacity of the illustrious author in recommending a practice which, though he seems never to have had an opportunity of adopting it himself, has yet been attended with the happiest results in the hands of others who no doubt acted on Sir A. Cooper's authority. Thus Mr. Bloxam (Med. Gaz., 1833, vol. xii., p. 702,) records a case of dislocation on the pubes with fracture of the femur reduced on the eighth day. The most remarkable case of the kind with which we are acquainted occurred in the practice of M. Etene, (Gaz. Méd. de Paris, 1838, p. 751:) A man was struck down by the fall of a tree; the left femur was dislocated and fractured about its centre, there was a penetrating wound of the left knee-joint, and a compound fracture of the left fibula, with dislocation of the corresponding ankle; Sir A. Cooper's practice was adopted in this case, the dislocation was readily reduced, and the man ultimately did well.

Sir A. Cooper's opinion as to the period within which reduction of dislocations may be safely attempted is of course generally known: he considers "that three months for the shoulder, and eight weeks for the hip, may be fixed as the period at which it would be imprudent to make the attempt at reduction, except in persons of extremely relaxed fibre, or of advanced age." (p. 28.) On this passage, the editor has, in our opinion, a very judicious note. He asks whether there is not a better criterion to determine the point in question than mere length of time:

"Should not the principal consideration be the precise condition of the new joint, especially as to the degree of motion of which it is capable, for by this a fair judgment may be formed as to what extent nature has altered the surfaces of the bones in contact to fit them for the functions of a joint in their new situation. If any useful motion can be performed, then I believe it may be considered as ill judged to attempt to restore the dislocated bone to its former articulating cavity, for it seems invariably to happen that as a new joint becomes fitted for use, so the structures of the old one are rendered incompetent to restoration."

These changes, the editor conceives, depend less on time than on the condition of the limb as to rest or motion: if the limb has been kept motionless, the changes occur very slowly; if continued efforts have been made to use the limb, they are apt to occur more rapidly; and once any useful degree of motion has been acquired, the editor deprecates any attempt at reduction.

When we consider that some dislocations—say of the femur—have been reduced after four, six, eight or even eighteen months, while, on the other hand, in most cases reduction is impossible after the lapse of a few weeks, it is difficult to avoid the conclusion that some difference must exist in the pathological condition of the parts in two classes of cases differing so widely in their results. We think it extremely probable that Mr. B. Cooper has the merit of suggesting at least one of these differences, and he has also probably indicated the signs which will aid our practice, by assisting us to diagnosticate the condition of the parts. Should experience confirm his opinion, he will have made a very valuable contribution towards the solution of a most important difficulty in one of the great questions of surgery. The opinion of M. Malgaigne on this point being, to a certain extent, borne out by pathological anatomy, is worthy of being placed in juxtaposition with Mr. B. Cooper's explanation of this important and difficult question. He considers that whenever the capsular ligament is torn to but a moderate extent it still continues to secrete synovia, which keeps open the passage of communication between the articular cavity and the head of the bone in its new situation, which will therefore readily regain its natural position by traversing the passage thus kept open by the synovia, if the adhesions it has formed be once broken up. When, however, the capsule of the joint is very extensively lacerated the articular cavity becomes filled up, and reduction is impossible. We have as yet, however, got but little precise and positive knowledge on this important subject.

The case in which Dieffenbach effected the reduction of a dislocation of the shoulder, of two years' standing, by "the subcutaneous section of everything that resisted him," is added to the text of the present edition, (p. 29) without any comment or opinion as to the value or propriety of the practice.

*Dislocations of the hip-joint.* The anatomical description of the hip-joint (p. 34 *et seq.*,) appears to have been revised by the editor, and yet it requires still further correction on one or two points which have a most important bearing on the history of dislocations of the femur. No description is given of the acetabulum in the dry state. We are told that in the recent subject it "is much deepened by a ridge of fibro-cartilage, which surrounds its brim, and which fills up the notch on the inner side. Now the acetabulum presents three distinct notches or depressions of great importance in a surgical point of view, as it is the existence of these notches that determines the direction of the displacement of the femur in all ordinary cases, and explains why it is that the head of the bone is always found (save in very rare and exceptional cases) in one of four (or regarding the more ordinary cases, only in one of three) determinate and constant situations. Sir A. Cooper (p. 100) considers this remarkable fact "as the natural result of the influence of the muscles which drew the bone into these positions." But an attentive examination must convince every one, we conceive, that the configuration of the brim of the acetabulum is at least the chief cause of the phenomenon in question. The brim of the acetabulum is in point of fact very prominent, superiorly and inferiorly, in which directions displacement of the head of the femur is consequently exceedingly difficult to be effected. At about the union of the upper and middle thirds of its *anterior* border (we use this as a more proper phrase than *inner* border, as the acetabulum looks chiefly outwards, and but very slightly downwards and forwards,) is another elevation corresponding to the ilio-pectineal prominence above, and especially below which the brim of the cavity is to a certain extent deficient, and thus we have two distinct *anterior notches*, through either of which the head of the femur may escape. If it escapes through the upper of these depressions *dislocation on the pubis* is the result; if through the lower one, dislocation into the *thyroid foramen* ensues. The *posterior notch* is the largest; it occupies almost the entire length of the *posterior margin* of the acetabulum, and its depth is perhaps one third of the entire depth of this articular cavity. Hence *one reason* of the facility and frequency of the occurrence of dislocation backwards. No doubt in the recent state, the cotyloid ligament fills up these depressions, but it is scarcely necessary to say that it cannot resist displacement as effectually as a bony ridge would. Sir A. Cooper (p. 100) says,

"There seems something a little anomalous in the frequency of the dislocation upwards and backwards upon the dorsum of the ilium; for upon examination of the hip-joint it will be found that it is the direction of all others in which there seems to be the greatest protection against dislocation, the capsular ligament being there the strongest, the edge of the acetabulum the most elevated, and the ligamentum teres offering the greatest hinderance to displacement in that direction. There is comparatively a much greater apparent facility to the dislocation into the foramen ovale, for the under and inner part of the acetabulum is partly formed of fibro-cartilage; the capsular ligament is much thinner here than at the upper part; and the ligamentum teres does not offer the same resistance to the displacement in this direction that it does to that over the upper part of the cotyloid cavity."

It is to be observed, that Sir A. Cooper seems to have applied his mind but little to the *mechanism* of dislocations. In the 4to edition there is, we believe we may say, not a syllable on this subject; the chapter from which we have made our last extract not appearing in the earlier editions. The difficulty here started is easily explained, and all the common dislocations of the hip can be not only accounted for, but it can be shown that they are the *only* dislocations which *ought* (if we may so speak) to happen with facility. We cannot, of course, enter into this discussion at length; suffice it to say, that the explanation chiefly flows from the anatomical considerations already detailed, coupled with the fact, that two of the most extended motions of the thigh are precisely those which approximate the head of the femur to the depressions that exist in the brim of the acetabulum. If the most frequent displacement of the femur was *upwards* its occurrence would be indeed anomalous, but the direction of the dislocation is upwards *and backwards.* We leave it to our readers to judge, whether the discussion at which we have here glanced did not come fairly within the scope of the editor's duty.

*Dislocation of the hip-joint on the dorsum ilii.* In the original 4to edition we find the following passage:

"In the dislocation upwards, the pyriformis and the glutei muscles are all shortened, as are also the triceps and pectineus, the psoas magnus and iliacus internus, the rectus, the semitendinosus, and semimembranosus, and one head of the biceps. The obturator externus is shortened; but the obturator internus, gemini, and quadratus are put upon the stretch."

It is very remarkable that in all the subsequent editions this passage has been expunged, without the true pathological anatomy of the accident being substituted in its stead. This is a deficiency which we certainly think the editor should have supplied.

The following valuable case of fracture of the acetabulum is inserted in the present edition (pp. 57-8) amongst the cases of dislocation on the dorsum of the ilium, and perhaps properly so, as the two accidents resemble each other very much.

A strong, young, muscular man, aged twenty-seven, was admitted into Guy's Hospital, September 2d, 1839, under Mr. Key. Shortly before admission he had been struck by a three ton weight on the back of the right leg and thigh in such a manner as to force the right knee in advance of and across the other leg.

"When the patient lay on his back the right leg appeared shortened two inches; the knee in advance of the other; the foot inverted, and lying over but not in contact with the dorsum of the left foot; the trochanter was unusually prominent, and nearer the anterior superior spinous process of the ilium than natural; and the anterior inferior spinous process could distinctly be felt. The patient had considerable motion of the hip, and could in some degree bend the knee, but could not evert it. After some exertion he could, when standing, bring the heel of the right foot within an inch of the ground, the toes remaining inverted. By employing gentle extension the limb could be elongated to its proper dimensions, and the prominence of the trochanter was considerably diminished, but returned as soon as the extension was discontinued. On rotating the femur with one hand applied to the knee, a very distinct crepitus could be felt at the hip. The crepitus could be felt only by the hand applied to the knee, not being discovered when the hand was placed on the hip. The trochanter on rotation moved with the femur, which could be bent and inverted, but could not be so much everted as to turn the toes out. The head of the femur could not be felt on the dorsum ilii."

Mr. Morgan attempted reduction with the heel against the perineum. On making very moderate extension the head of the bone returned into the acetabulum "the return was accompanied with a jerking, grating motion not with the ordinary snap," and as soon as extension was discontinued the head of the bone slipped from the acetabulum. He was placed with both legs extended and bandaged together, but the hip becoming painful, hot, and swollen, was leeched and the limb was placed on a double inclined plane. It is an extraordinary instance of carelessness or of oversight that the termination of this interesting case is not given. The last statement respecting it is that on the 25th September, 1839. "He still continues on the plane; there is slight shortening and inversion of the foot; he can evert the foot, but not the knee; there is no pain in the hip, and the joint has its natural shape." This case occurred in the hospital of which the editor is himself one of the surgeons. He might have learned, one would think, the result of the case for the trouble of asking, and yet he leaves us in ignorance as to its issue.

At pp. 51-2 the editor inserts two cases of dislocation on the dorsum ilii, communicated to him by Mr. Elliott, stating that "they show that an able surgeon may succeed, even with inferior means, if he has the right principles to guide him." The ability exemplified in these cases simply consisted in reducing the dislocations without pullies, and by the aid of a sufficient number of vigorous soldiers. Surely Mr. Cooper ought to know that hundreds of dislocated hips have been reduced by the hand; and that this has for many years been the mode adopted, by preference, on the continent.

*Dislocation into the foramen ovale.* With reference to the treatment of this accident, Sir A. Cooper, in the 4to edition, (pp. 37-8,) objects to the mode of reduction employed by Mr. Hey, but most candidly adds,

"I am not sure that, in all respects, I understand the description of the method which he adopted." The entire passage is omitted in the present edition; and we must say, that, in our opinion, the editors should have restored it to the text. The materials for the history of surgery should all be preserved in the most authentic form. The very passage, for example, just adverted to is of great importance when we come to *weigh authorities* respecting the most eligible mode of reducing dislocations of the femur—a point far indeed from being yet definitely settled.

*Dislocation into the ischiatic notch.* With respect to the diagnosis of dislocation into the ischiatic notch, the editor adds the following note:

"I have heard it a matter of discussion between equally competent surgeons respecting a case of dislocation, whether the head of the bone was placed upon the dorsum ilii or in the ischiatic notch; the dispute arising as to the extent of the shortening of the limb, which, in my opinion, does not form a very perfect diagnostic mark; for the limb becomes invariably shorter as the space of time increases since the accident has occurred, so that it may vary from half an inch at first to a subsequent shortening of three inches. The best sign of this accident, I believe, is the greater displacement of the head of the thigh-bone backwards, and the consequent obliquity of the shaft, so as to direct the knee across the middle of the opposite thigh, just above the patella." (p. 72.)

We must certainly object to the statement, that the head of the femur, if in the ischiatic notch, can be drawn upwards two inches and a half and still continue in that space. No doubt, the extent to which the limb may be elongated varies considerably, and we do not deny that the tonic contraction of the muscles exerts some little influence in this respect. There is, however, an anatomical circumstance which explains the variability of this symptom in different cases. The relative height, as Malgaigne observes, of the cotyloid cavity and of the ischiatic notch must evidently vary with the angle of inclination of the pelvis; but this angle does vary, both in the two sexes and in different individuals of the same sex. We have not space, nor is it indeed our business, to follow out these considerations, which evidently have a very important bearing on the symptomatology of this dislocation.

*Dislocation on the pubes.* Two of the cases recorded in this chapter are interesting because of the unusual situation of the head of the femur; which in one case, (pp. 89-90,) "was placed upon the femoral artery so as to stop the pulsation." While in the other case, "it was placed to the inner side of the femoral artery."

*Anomalous dislocations of the hip-joint.* In the earlier editions, Sir A. Cooper, when treating of *varieties of dislocations* of the hip-joint, admitted of but four dislocations of the femur; those in fact which we have just so briefly passed in review. The only other dislocation which he even mentions is that "downwards and backwards," respecting which he speaks somewhat vaguely, but on the whole seems to deny the possibility of its occurrence. In the quarto edition, (p. 33,) he does not absolutely negative its possibility, "yet," he says "I am disposed to believe that some mistake has arisen upon this subject." Again, (4to, p. 72,) he says "I cannot help thinking that some anatomical error must have given rise to this opinion, &c.;" and finally, (4to, p. 76,) we read, "it is to be remembered, that there is no such accident as a dislocation of the hip downwards and backwards." In the present edition the three passages just quoted are expunged, and the first of the foregoing citations is replaced, (p. 36,) by the following sentence: "Besides these four, two other anomalous dislocations have been described, of which we shall speak in the proper place." Sir A. Cooper's opinions, therefore, on this subject underwent considerable change, a circumstance, however, which no reader could even suspect from the perusal of the present edition.

In the present chapter (on anomalous dislocations, p. 100 et seq.) Sir A. Cooper admits the possibility, and describes cases of dislocation of the femur upwards and forwards—upwards and downwards. He considers such accidents, however, as anomalous, and as only capable of occurring when the dislocation is complicated with fracture, or when collapse from any cause is present, when the sheer physical force which produces the accident may place the bone in some unusual position, which it maintains either from a portion of the broken bone resisting its being acted on by the muscles, or from the muscles themselves having lost all power. This opinion, we need scarcely remark, is very questionable, and is far from being generally assented to. We would have been glad had the editor discussed this interesting point, sufficient materials for the consideration of which would have been afforded by the cases detailed in the present volume, together with those which are recorded elsewhere. The editor should also, we think, have at least noticed the other observed varieties of these "anomalous" dislocations not mentioned in the text. We should suppose that the case of dislocation on the spine of the ischium observed by Earle, for example, (Lancet, vol. xi. p. 159, 1827,) must have been within his knowledge.

The dislocations recorded in this chapter being of extremely rare occurrence, we shall abstract two of the number.

*Dislocation of the hip upwards.* A man was admitted into Guy's hospital, having about an hour previously fallen backwards while helping to carry a heavy crate down stairs. He fell backwards, receiving the weight on the groin. When he lay extended on his back,

"The left leg was shortened at least two inches, and the foot excessively everted, so as almost to give the toes a direction backwards. The injured limb had a tendency to cross the sound one, so as to throw the heel of the former over the instep of the latter; nevertheless, when they were placed side by side they remained in that position. The leg was susceptible of all the natural motions to some extent, with the exception of rotation. The projection of the trochanter major was entirely lost, while the luxated head of the bone could be felt under Poupart's ligament, just below and to the inner side of the anterior inferior spinous process of the ilium, and the junction of that bone with the pubis. It thus rested upon the brim of the pelvis, and projected towards the abdomen. The femoral artery was not displaced, but could be traced on the inner side of the bone."

The mode of reduction adopted is worthy of notice. Extension was made downwards from the knee by means of a towel; while Mr. Morgan fixed the pelvis by sitting on the bed and placing his foot between the scrotum and the thigh. Three or four students made steady extension for about three minutes; the patient was then directed to raise his shoulders from the bed, and the thigh was forcibly rotated inwards, the power of a long lever being obtained by bending the knee at right angles to the thigh, and grasping the knee in one hand and the foot in the other. The head of the bone returned immediately with a snap into its socket. (pp. 103-5.)

*Dislocation downwards.* Mr. Keate was called to see a gentleman whose horse had fallen with him and upon him into a deep and narrow ditch. The limb was at least three or three and a half inches longer than the opposite one. The thigh was much flexed upon the pelvis, the leg as much bent upon the thigh. The whole limb was carried outwards or apart from the other to a greater extent than Mr. Keate had ever observed in a case of luxation. The head of the femur was inferior to the ischiatic notch, and was found lying close to, and on a level with, the tuberosity of the ischium. In the first attempt at reduction the head of the femur was thrown into the foramen ovale; a second extension enabled Mr. Keate to place it *nearly* in its proper position in the acetabulum, but it could not be perfectly replaced; and on gently moving the bone a distinct grating as if of ruptured cartilage was heard. By drawing the upper part of the femur outwards, and pressing the knee sharply inwards, the head of the bone was replaced with a snap in the acetabulum. But even then, Mr. Keate says, "I was enabled to elongate or pull down the limb, and it was evident to me that this was owing to a portion of the cartilaginous labrum having been broken off."

Mr. Cooper has not taken any notice of the great difference of opinion that at present exists respecting the general principles that should be adopted in the reduction of dislocations of the hip. Many practitioners are of opinion that in most if not in all displacements of the femur, the thigh should be flexed on the pelvis, and the leg flexed upon the thigh; and independently of the cases recorded by the older practitioners, as Pouteau, Pott, Hey, &c. numerous examples are being constantly published in which these accidents have been reduced with the greatest facility by adopting this plan. Several of the cases Mr. Cooper has edited illustrate the—at least occasional—success of this old and recently revived method, (e. g. case lxiii. p. 96-7;) and would have afforded opportunity for interesting and valuable comment.

Our observations on the remainder of the present volume must be rapid and general. The chapter "on Fractures of the Pelvis" is newly arranged, contains some new matter of interest, and its practical value is decidedly increased; it calls, however, for no particular remark on the present occasion. The chapter "on Fractures of the upper part of the Thigh-bone" has also gained much in clearness and precision from the rearrangement of the matter, and we are happy to be enabled to add from the editorial comments. Almost every point requiring illustration has been noticed by the editor; and, for the most part at least, in a satisfactory manner. At p. 131 the editor in a note alludes to that puzzling phenomenon the inversion of the limb that occasionally occurs in fracture of the neck of the thigh-bone completely internal to the capsular ligament. We could perhaps have wished his observations on the subject to have been somewhat fuller; but, after all, the note possibly is a fair summary of the state of our knowledge or rather ignorance on the matter. As to the symptoms of this accident the editor (p. 133,) properly adds to those detailed in the text the following note:

"Another strong diagnostic mark of this accident may be ascertained by desiring an assistant to make extension of both limbs and simultaneously rotate them, when the surgeon, by placing his hands upon the trochanters, will perceive that they move in the arcs of different circles, that on the injured rolling on its own axis, while the healthy trochanter describes an arch of which the neck forms the radius." The editor adds, "and further it will be found that the patient cannot raise the whole limb from the bed, in consequence of the thigh-bone having lost its *point d'appui* in the acetabulum."

This latter observation is no doubt true in the immense majority of cases, but the editor should have warned his junior brethren that in some rare instances a patient labouring under this accident can not only raise the limb from the bed but even walk for some time after the receipt of the injury. As to the age at which fracture of the cervix femoris within the capsular ligament is liable to occur, Sir A. Cooper, as is well known, had in all his immense experience "*only known two cases occur under fifty years of age.*" The editor (p. 135,) adds to these "Mr. Stanley's case, in a boy aged eighteen, reported in the Med. Chir. Trans. vol. xviii."

We cannot be expected to enter into the questio vexata of bony union of fractures of the neck of the thigh-bone within the capsular ligament; we believe that Sir A. Cooper's views, long misunderstood or misrepresented, are now assented to by the great majority of surgeons. At pp. 144 and 155 we have two notes from the editor on the subject, the one being pretty nearly a repetition of the other. In the latter the editor says, "I am quite of Sir A. Cooper's opinion, that fractures of the neck of the thigh-bone within the capsular ligament do not, excepting under peculiar circumstances, unite by bone," &c.: and he further says, "I maintain that there are several circumstances tending to prevent the ossific union of a fracture of this part of the thigh-bone," &c. Of the several circumstances thus "maintained" by the editor the only one of any importance not directly mentioned by Sir A. Cooper, is the peculiar change sustained by the cervix femoris in advanced life; and which, as it renders it more fragile, may, Mr. Cooper argues, be fairly considered as rendering it, when broken, incompetent to set up restorative action. This reason for non-union has been long since assigned by Velpeau and others (atrophie senile—imbibition huileuse), to whom, however, the editor makes no reference.

We pass over the chapter "on Dislocations of the Knee." In the chapter "on Dislocations of the Head of the Fibula," the editor gives a case of this exceedingly rare accident. Sir A. Cooper had already recorded one case, but it was connected with compound fracture of the tibia. In Sanson's case it could be scarcely said that dislocation existed, the ligaments of the articulation were ruptured, and the head of the bone could be readily displaced by pressure, but when left to itself it resumed its natural position. Mr. Cooper's case is, however, an unexceptionable example of dislocation of the upper extremity of the fibula from violence. The accident occurred from a waggon passing over both limbs.

"The left fibula, when traced upwards, seemed to pass more backwards than natural. It was also more moveable than usual, and the upper part of the bone was felt in the popliteal space. There was little deformity visible, but the projection of the outer head of the soleus muscle was not quite so distinct as in the other leg..........There was scarcely any pain given by motion of the limb, and the man possessed the power of extending it perfectly and of flexing it to a considerable extent.... .......The dislocation was reduced by bending the leg to relax the biceps muscle, and then grasping the head of the fibula and pressing it into its natural situation."

Some difficulty was experienced in maintaining the bone in situ, to effect which object a tourniquet pad was applied over it for nearly two months, at the " end of which time the fibula remained rather behind its usual situation, but was perfectly fixed."

In the chapter " on fractures of the patella," we find two new cases (one from the editor's practice) of compound fracture of the patella terminating favorably. And the editor has also inserted from the fifth vol. of Guy's Hospital Reports, Mr. Ward's most remarkable case of recovery from a gun-shot which carried away nearly the entire of the patella.

The chapter " on dislocations and fractures of the ankle-joint," admirable as it is, need not detain us on the present occasion. It is true that it is improved in form and general arrangement, and is enriched with some new and valuable cases, but still it would not be easy to specify the improvements within any moderate space, nor would it perhaps be very useful to do so; as it does not in point of fact *essentially* differ from the corresponding chapter in former editions. This chapter has called forth scarcely a remark from the editor. We shall only observe that the author, in simple dislocation of the tibia inwards (p. 253,) and also in fracture of the fibula near the ankle-joint (p. 317,) recommends almost exclusively that the leg should be placed on the outer side in a semi-flexed position. Dupuytren's mode of treating these cases is so very obscurely and imperfectly described, that we suspect few if any practitioners would be able thence to apply his method, even with the aid of the diagram, (p. 256.) The editor might, we conceive, have advantageously appended a note on this subject, and at the same time have given an account of the mechanism of these injuries as described by Dupuytren.

We pass over " dislocations of the foot," and " dislocations of the jaw." As regards " dislocations of the clavicle," we have only to remark that the editor might have noticed M. Pellieux's case of dislocation of the sternal extremity of the clavicle backwards from violence, the accident having arisen from the patient falling under his horse. (Revue Médicale, 1834, p. 161.) The case of this affection recorded by Sir A. Cooper, long the only one known, was after all scarcely an example of dislocation in the common sense of the phrase, as it was the result of disease.

The chapter on "dislocations of the shoulder" shall not detain us long, both because of want of space and because the editor's annotations on it are very few in number and very unimportant. We may observe that, at p. 371, a case is given in which Dr. Gibson, of Pennsylvania, in endeavouring to reduce a dislocation of the shoulder, of two months' standing, effected indeed the reduction, but at the expense of tearing across the axillary artery, which caused the death of the patient. A reference to Dr. Gibson's work of surgery would have shown the editor that this lamentable accident occurred *three* times in the practice of that gentleman; who has, with the most praiseworthy candour, published the cases in detail as a lesson and warning to his professional brethren. At p. 379, the editor describes, and illustrates by an admirable engraving, Mr. White's method of reducing dislocation of the humerus, a method recently revived by M. Malgaigne, and in some instances with remarkable success. In the section " partial dislocations of the shoulder-joint," the editor has incorporated Mr. Soden's observations on dislocation of the biceps tendon from its groove.

We pass on to "Dislocations of the Radius," and cannot but regard it as singular that the editor makes no observation on the remarkable fact that Sir A. Cooper's experience respecting these dislocations differed so widely from that of every other surgeon who has written on the subject. Sir A. Cooper met with seven cases of dislocation of the head of the radius forwards; while he never saw dislocation of the radius backwards in the living subject. Setting Sir A. Cooper's experience aside, the number of recorded cases of dislocation of the radius forwards are extremely few, while we have at hand upwards of twenty references to dislocation of the radius forwards. Two of these observed by Professor Langenbeck are incorporated with the text. (p. 460.)

We have already almost exceeded our limits, but we have fully accomplished the object we had in view, which was not to give anything like a detailed analysis of the present volume, but to enable our readers to judge of its value as compared with the former editions of the same work. In so doing it will be perceived that we have not scrupled to express our opinions freely and to find fault where we considered there was cause for so doing. It is to be observed, however, that any strictures we have made apply rather to faults of omission than of commission. Had the editor confined himself to merely producing the text of his great original, our duty would have been to deal out unmixed praise and thank him for an edition decidedly superior to any of its predecessors; but he made his option to publish an annotated edition, and in so doing we think he judged most wisely. The distinguishing merit of the original work is its eminently practical character; and one of its most distinctive peculiarities is the almost complete absence of reference to books or to the experience of others, always excepting such cases as were originally and directly communicated to the author himself. This great work, however immense its value, was still but the record of the experience of one man and of a few individuals of *his school;* and it is therefore obvious that its merit was susceptible of being augmented in no trifling degree by judicious comments and additions from the experience of others, who had been set in the track of profitable observation by the impulse communicated by its admirable contents. Mr. Cooper, then, as we think, most judiciously chose the part of publishing an edition with notes, and consequently professes to criticise and comment on the text—to correct erroneous doctrines—to supply defects—to make the work, in a word, represent the existing state of knowledge respecting the subjects of which it treats. Such being the case, we thought it right to examine the performance not simply with reference to what the editor has done, but also with a view to what he might and, as we think, should have done.

If, however, we look at the present edition merely as it is—if we take it as it is given to us, we feel bound to say, that it is a most valuable and important addition to surgical literature. In it we find the last, the most matured views of its venerable author, who, with unexampled zeal, continued to almost the last moment of his life to accumulate materials for perfecting his works. Every practical surgeon must add the present volume to his library. Its commodious and portable form—no mean consideration—the graphic, the almost *speaking* force of the unequalled illustrations, the copious addition of valuable and instructive cases, and the great improvement in clearness and precision which has been gained by the judicious arrangement of the materials, all combine to render the present edition indispensable. The woodcuts profusely scattered through almost every page are beyond praise: nothing can exceed their faithful accuracy and clearness. Truly is it remarked by the editor that "the advantages of such engravings being placed in immediate connexion with the portion of the text which they are intended to elucidate, will not pass unnoticed by those who have felt the inconvenience of having to search at the end of the volume to which reference occurs in the text;" and the benefit of such an arrangement is vastly enhanced when the illustrations are of such exquisite beauty as those of Mr. Bagg.

---

